# Pleomorphic Adenomas and Their Atypical Morphology: Pitfalls in the Diagnosis of Salivary Gland Tumors

**DOI:** 10.3390/diagnostics16142168

**Published:** 2026-07-11

**Authors:** Alexandra Corina Faur, Alina Maria Șișu, Codruta Ileana Petrescu, Aura Jurescu, Camelia Vidiţa Gurban, Daniela Cornelia Lazăr, Diana Andrei, Sorin Lucian Bolintineanu, Laura Andreea Ghenciu

**Affiliations:** 1 Department of Anatomy and Embryology, “Victor Babeș” University of Medicine and Pharmacy Timisoara, Eftimie Murgu Square, No. 2, 300041 Timisoara, Romania; faur.alexandra@umft.ro (A.C.F.); petrescu.codruta@umft.ro (C.I.P.); s.bolintineanu@umft.ro (S.L.B.); 2 Department of Microscopic-Morphology-Morphopathology, ANATPATMOL Research Center, “Victor Babeș” University of Medicine and Pharmacy Timisoara, Eftimie Murgu Square, No. 2, 300041 Timisoara, Romania; jurescu.aura@umft.ro; 3 Department IV Biochemistry and Pharmacology, Discipline of Biochemistry, “Victor Babeș” University of Medicine and Pharmacy Timisoara, 300041 Timisoara, Romania; gurban.camelia@umft.ro; 4 Department V Internal Medicine I, Discipline of Internal Medicine IV, “Victor Babeș” University of Medicine and Pharmacy Timisoara, 300041 Timisoara, Romania; lazar_daniela@yahoo.com; 5 Department of Balneology, Medical Rehabilitation and Rheumatology, “Victor Babeș” University of Medicine and Pharmacy Timisoara, 300041 Timisoara, Romania; andrei.diana@umft.ro; 6 Department of Functional Sciences, “Victor Babeș” University of Medicine and Pharmacy Timisoara, Eftimie Murgu Square, No. 2, 300041 Timisoara, Romania; bolintineanu.laura@umft.ro; 7 Centre for Translational Research and Systems Medicine, “Victor Babeș” University of Medicine and Pharmacy Timisoara, 300041 Timisoara, Romania

**Keywords:** mixed tumors, atypical pleomorphic adenoma, benign salivary gland tumors, atypical morphology, invasion, metaplastic changes

## Abstract

Pleomorphic adenomas (PAs) are benign mixed tumors of the salivary glands that usually present with morphological characteristics consisting of epithelial and myoepithelial cells and a chondromyxoid stromal component. Atypical cells; squamous, osseous, lipomatous, endocrine-like or apocrine metaplasia; aspects of invasion (vascular, perineural, in adjacent tissues or the capsule); necrosis; and unusual dimensions were reported as uncommon aspects that can be encountered in PAs. This study consisted of a retrospective analysis of the morphological characteristics of PAs, aiming to identify their unusual features and prevent misdiagnosis. We discuss the diverse morphological aspects of PAs, with particular emphasis on the challenges associated with their diagnosis.

## 1. Introduction

Pleomorphic adenomas (PAs) are benign mixed tumors, consisting of epithelial and myoepithelial cells and mesenchymal elements and characterized by a variety of cellular and architectural aspects. The diversity of these tumors is not limited to morphological characteristics. PAs may be found in uncommon locations; they can show rare clinical aspects, unpredictable evolution and features that make them difficult to differentiate from malignant tumors of the salivary glands [1,2,3,4,5,6,7].

PA is the most common type of salivary gland tumor. In 60–85% of cases, these tumors are located in the parotid gland and only 5% in the submandibular gland. Also, approximately 10% of PAs affect the minor salivary glands of the palate, lip, oral floor, tongue, tonsil, pharynx, retromolar area, and even the nasal cavity, paranasal sinuses, trachea or larynx [1,2,3,4,5,6,7]. Cases have also been reported in glands with similar morphological structure such as the eyelids or other regions of the orbit like PA of the lacrimal glands [5]. PAs arising from the oral mucosa at the base of the tongue are estimated to account for less than 1% of minor salivary gland tumors [8]. Cutaneous PAs are more commonly known as chondroid syringomas of uncertain origin, and current data show that they have an eccrine or apocrine etiology. PAs of the ceruminous glands of the external auditory canal have also been reported [1,9]. The first case of nasal PA was reported in 1929 by Denker and Kahler [10,11,12]. Most cases of nasal PAs have been localized in the minor salivary glands of the nasal septum mucosa [11,12]. Eighty percent of parapharyngeal area tumors are benign, most of them with a salivary gland origin (40–50%), PA being the most common histological type [13]. Parapharyngeal PA can develop de novo from minor salivary glands of this region or from the deep lobe of the parotid gland [14]. Another unusual localization where PAs can develop is the pterygopalatine fossa [15].

PA is more frequently reported in females, typically during the fourth to sixth decades of life, with a mean age at presentation of approximately 46 years. However, their incidence varies widely, with cases reported of tumors identified in the pediatric population, in the first decade of life and also PAs diagnosed in the 10th decade of life [5,16,17,18,19]. Tumor recurrences are reported in patients older than 30 years of age [3].

Clinically, PAs are slow-growing masses that reach 2–4 cm in size at the time of excision. Small tumors typically form smooth, mobile, firm tumor nodules, but large tumors have numerous protuberances and may push the overlying skin or mucosa, compressing the capsule and focally extending into adjacent tissue [16,17]. However, cases of PAs with a long clinical course of up to 30 years and weights ranging from 1 to 27 kg have been published [16,17,20]. Also, in cases with multifocal tumor recurrences, tumors may present as fixed masses [16,17,18,19]. Although rarely present, facial paralysis and pain have been reported more frequently in cases with infarcted tumors. In the minor salivary glands of the palate, tumors usually belong to the mucosa located at the junction with the soft palate and are generally well fixed due to the proximity of the mucoperiosteum [16,17,20]. In the literature, cases of PA have been reported that were accompanied by bone remodeling or, in cases with tumor recurrences, even bone destruction or malignancy. These aspects have been seen mostly in patients with PAs developed in the minor salivary glands of the nasopharyngeal mucosa, lacrimal glands or ceruminous glands of the auditory canal [21,22].

The malignant potential of PA has been studied, raising numerous questions. In the literature, the term mixed malignant salivary gland tumor encompasses three tumor entities that should not be confused: carcinoma ex pleomorphic adenoma (CEPA), carcinosarcoma and metastatic mixed tumor [16,17,23]. Metastatic PA (MPA) belongs to a group of rare tumors that are histologically identical to PA but spread to distant or regional sites [23]. It has also been proposed that the presence of atypical cells in some PAs warrants the term focal carcinomas for these aspects [24]. “Bizarre” cells have likewise been reported in pleomorphic adenomas [2]. The term atypical PAs has been proposed for tumors that, although benign, show minimal nuclear atypia and rare mitoses [25]. Recent data report PAs that erode the adjacent bone or invade the tumor capsule, the extracapsular vessels and nervous structures and are defined as atypical features for PAs [26,27]. Morphological features such as the presence of tyrosine crystals and foci with atypical (dysplastic) cells are also identified [16,17,28]. The evolution of the above features must be known and followed-up.

In this narrative review, we discuss the diverse morphological aspects of PAs, with emphasis on the challenges in their diagnostic process. Our study evaluates the specific morphological features of PAs, with the objective of identifying unique, unusual or atypical aspects useful for the diagnosis of uncommon entities and to assess the role of tumor morphology in relation to their evolution.

## 2. Materials and Methods

This study conducted a retrospective analysis of morphological aspects of PAs, aiming to identify unusual features and prevent misdiagnosis. A literature search of English language articles was carried out using Google Scholar, Scopus and PubMed databases with the purpose of identifying studies about PA morphology. The terms used for the search were “mixed tumors”, “atypical pleomorphic adenoma”, “benign salivary gland tumors”, “atypical morphology”, “invasion” and “metaplastic changes”. The inclusion criteria were: case reports and studies of PAs, case reports/studies about atypical PAs, review reports with unusual features of PAs, reports documented in the English language. The exclusion criteria were: reports documented in languages other than English, articles not concerning PAs, articles with other diseases of the salivary glands, reports with insufficient data.

A total of 142 papers were selected based on the inclusion criteria. After reading the abstracts, 108 were chosen and a further selection was made by reading the complete articles. A final selection of 100 articles was made according to the aforementioned criteria. After finishing the selection of articles, the papers were analyzed by the authors. The articles were divided into two categories: one contained the newest reviews and studies on current knowledge in assessing the morphology of common PAs (62 studies) and the other (38) included studies that explored atypical PAs. The following data were extracted: 1. for typical PAs—the frequently reported clinical–morphological aspects; and 2. for PAs reported as atypical—demographical aspects, symptoms, invasion, gross aspects, atypical microscopic features (metaplasia/cells reported as atypical/tyrosine crystals) and follow-up period. A database with PAs reported as unusual or atypical was created and further reviewed by the authors.

**Figure 1:**
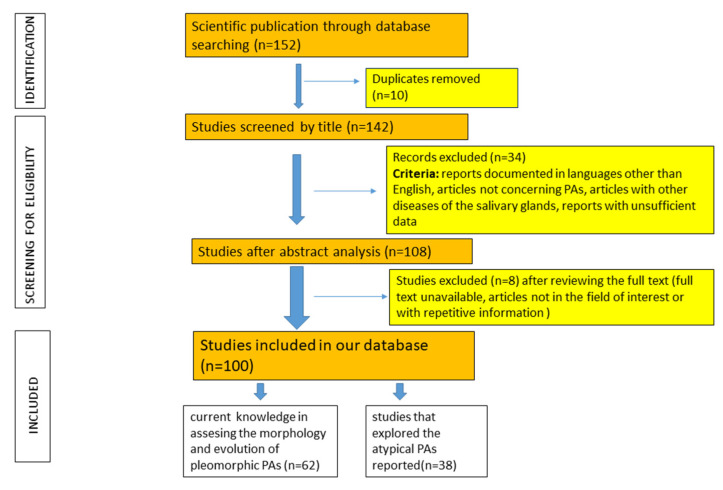
The flowchart of the algorithm used for the article selection.

The PRISMA criteria were consulted and adapted for this study [29]. A flowchart of the literature search for this narrative review selection is shown in Figure 1.

Usually, the studies that reported PAs described a frequent morphology for these tumors represented by a cellular component consisting of a mixture of epithelial and myoepithelial cells arranged in ductal structures, aggregates, isolated or in solid areas in a chondromyxoid stroma, with variable proportions. This morphology is considered pathognomonic for the benign mixed tumor/classical morphological variant of PA. A total of 38 articles reporting rare and unusual aspects in PAs were identified [20,26,27,28,29,30,31,32,33,34,35,36,37,38,39,40,41,42,43,44,45,46,47,48,49,50,51,52,53,54,55,56,57,58,59,60,61,62,63]. Thus, 863 cases of PAs classified by the authors of the studies as being atypical were analyzed. The morphological features of atypical PAs were found in tumors that presented, in addition to the morphology of classic PA (CPA), aspects such as atypical cells; squamous, osseous, lipomatous, endocrine-like or apocrine metaplasia; aspects of invasion (vascular, perineural, in adjacent tissues or the capsule); necrosis; and unusual dimensions. Most of these atypical PAs were identified in women (531 cases). In 93.97% of patients, these tumors developed in major salivary glands, with 87.02% of cases located in the parotid gland, 6.97% in the submandibular gland and 6.02% in the minor salivary glands. Necrosis (6 cases), bone (4 cases), vascular (1 case) or perineural (2 cases) invasion were reported only in isolated cases. Also, lipomatous, apocrine and osseous metaplasia were less frequently reported (between 0.3 and 0.7% of cases). The data from studies with atypical PAs are summarized in Table S1.

**Figure 2:**
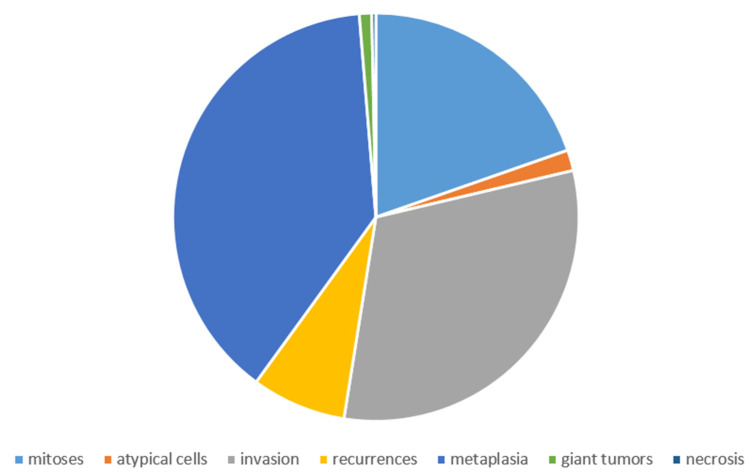
The distribution of analyzed cases [20,26,27,28,29,30,31,32,33,34,35,36,37,38,39,40,41,42,43,44,45,46,47,48,49,50,51,52,53,54,55,56,57,58,59,60,61,62,63] in which atypical aspects were reported.

**Figure 3:**
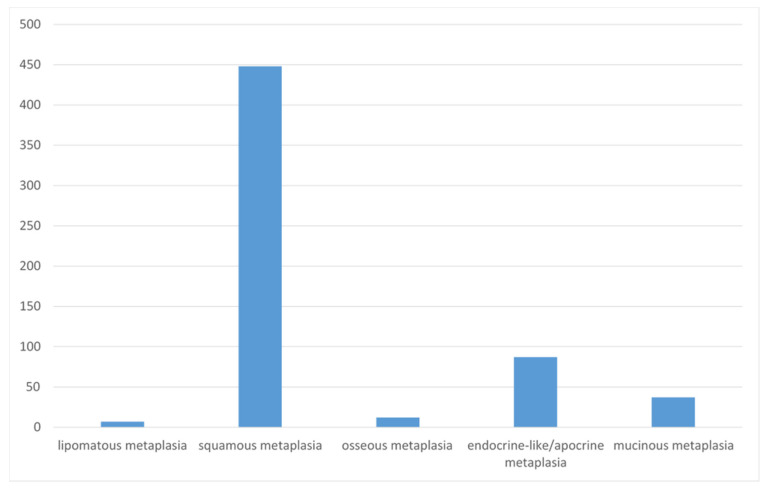
The distribution of metaplasia in the reported cases [20,26,27,28,29,30,31,32,33,34,35,36,37,38,39,40,41,42,43,44,45,46,47,48,49,50,51,52,53,54,55,56,57,58,59,60,61,62,63].

The morphology of PAs and the lesions to consider in their differential diagnosis are summarized in Table 1. Figure 2 and Figure 3 present the distribution of the atypical features and the metaplasia reported in the studied cases.

## 3. Clinical and Morphological Aspects of PAs

### 3.1. Typical Aspects Considered Pathognomonic for PAs

#### 3.1.1. Microscopical Aspects

**Figure 4:**
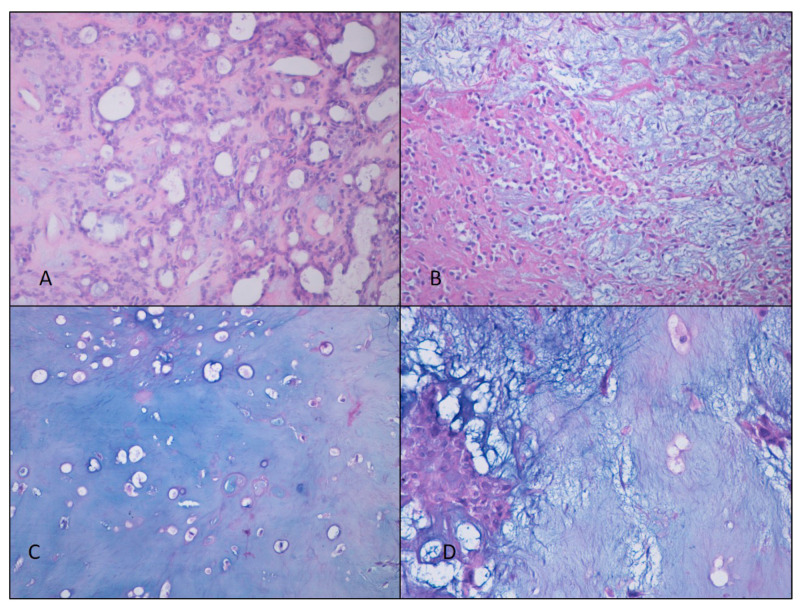
CPA with ductal, solid component and myxoid stroma (**A**,**B**), chondroid metaplasia (**C**) and chondromyxoid stroma (**D**). The images were obtained using a Leica DM750 microscope (leica microsystems (schweiz) ag max schmidheiny-strasse 201 ch-9435 heerbrugg switzerland) with a digital camera (200 and 400× magnification).

The World Health Organization (WHO) defines PAs as mixed tumors composed of epithelial and myoepithelial cells arranged in ductal structures, solid areas, cords or solid nests; in a stroma with fibrosis, hyalinization, myxoid areas and occasional chondroid areas (Figure 4); in variable proportions [16,17]. These features are found in most studies reporting cases of PAs and can therefore be considered pathognomonic for this type of tumor/classic PAs (CPAs) [16,17,64,65]. However, some CPAs may contain a more pronounced epithelial or stromal component, which makes differential diagnosis with other salivary gland entities or mesenchymal tumors difficult [66].

The epithelial component of a CPA is composed of columnar, cuboidal, polygonal, flattened, basaloid, plasmacytoid, fusiform, or clear tumor epithelial cells. The cells have large, vacuolar nuclei, some with prominent nucleoli and a low mitotic rate. These cells may be organized in solid sheets, trabeculae, cystic areas, or tubular ductal structures. The ductal structures, variable in size, are most often bordered by two cell layers—cuboidal luminal cells and, at the periphery, myoepithelial cells—which are either similar to luminal cells or with clear cytoplasm and angulated hyperchromic nuclei. The lumen of these ductal structures is free or contains eosinophilic material. When the epithelial component is dominant and represents the majority of the tumor, they are called cellular PAs [16,17,66].

The myoepithelial component of CPA most often completes both the epithelial and the stromal components, being represented by spindle, stellate or plasmacytoid cells. In most studies, cells with a spindle or plasmacytoid aspect proved to have an immunohistochemical myoepithelial origin. The myoepithelial cells may even form a fine reticular pattern or cords of spindle cells organized into palisades, forming a schwannoma-like aspect [67]. The spindle myoepithelial tumor cells are distributed in the epithelial component of the pleomorphic adenoma around the ductal epithelial structures, on their exterior or in richly cellular nests/areas. Myoepithelial cells can also be found isolated, in nests or in anastomosed plates and in the myxochondroid matrix of the tumor. These cells have elongated nuclei, with a slightly basophilic aspect and a blurred outline. The myoepithelial cells with plasmacytoid features are oval in shape and have a homogeneous hyaline cytoplasm, with round, eccentric nuclei [16,17,65].

**Figure 5:**
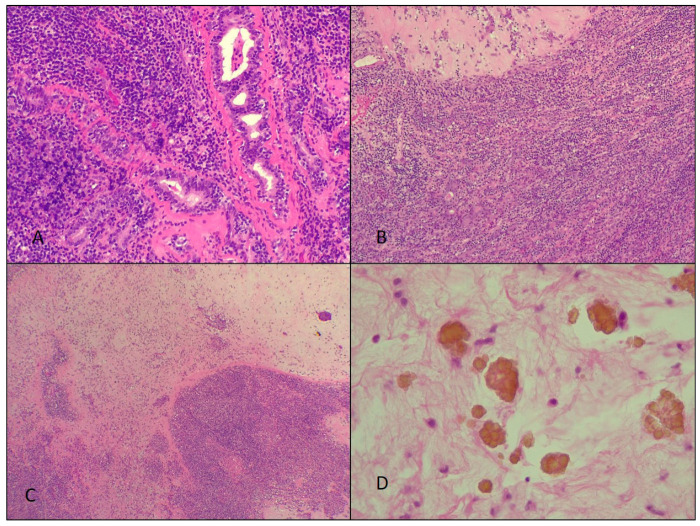
CPAs with lymphoid and chondromyxoid stroma (**A**–**C**) and CPA with tyrosine crystals (**D**). The images were obtained using a Leica DM750 microscope with a digital camera (200 and 400× magnification).

The stromal component of CPAs is represented by a mucoid/myxoid/fibrotic material and aspects of hyalinization, completed by fusiform or stellate myoepithelial cells, with areas of chondroid metaplasia [16,17,68]. The presence of a lymphoid component was also described in the stroma of salivary gland tumors [16,17,69]. Recent data quote PAs with abundant lymphoid stroma (Figure 5) [70,71,72]. Occasionally, tyrosine and oxalate crystals (Figure 5) may also be present [16,17,65].

Generally, the proportion of cellular or stromal elements is not important for the evolution of cases. However, according to some authors, PAs with a predominance of the myxoid component are an exception because, if not handled correctly during surgical excision, can cause ruptures of the tumor capsule, with accidental local implantation of their content, raising the risk of tumor recurrence [16,17,66].

PAs with classic morphology (CPA) and elements reported as part of their components are presented in Figure 4 and Figure 5.

Hybrid salivary gland tumors are less often mentioned. They are not defined as having atypical morphology. From the category of benign salivary gland tumors, hybrid adenomas with a component of canalicular adenoma and basal cell adenoma were cited: one case of basal cell adenoma, but with a malignant component represented by an adenoid cystic carcinoma, and another case of Warthin tumor and sebaceous adenoma, respectively [73,74,75,76,77]. The authors Seifert and Donath define hybrid tumors as a neoplastic lesion composed of two or more tumor entities belonging to a specific category, identical in origin, with simultaneous development in the same topographic region and encompassing a transition area between two or more components [74]. For PAs, unusual patterns of growth were described but are not listed as hybrid or atypical tumors. Thus, a canalicular-like growth pattern was recently reported in PAs. In the canalicular adenoma-like subtype of PAs, morphological aspects of CPA are described, with areas similar to canalicular salivary gland adenoma [70,78,79,80].

**Figure 6:**
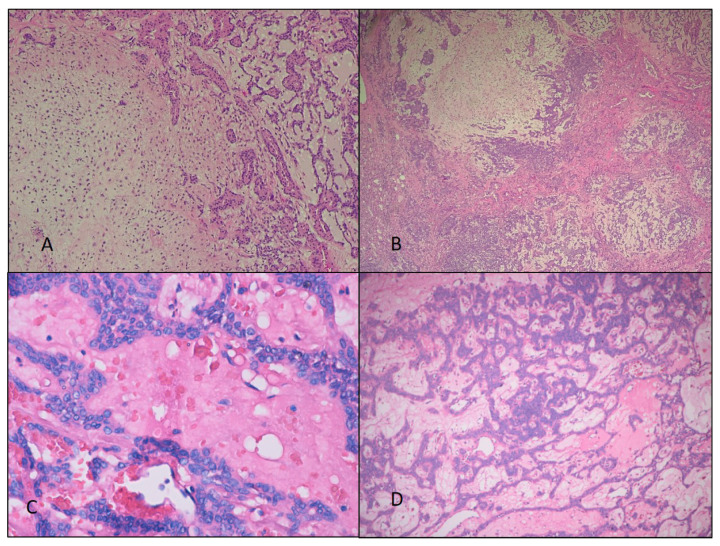
Canalicular adenoma-like PA with typical chondromyxoid stroma (**A**,**B**) and classic canalicular adenoma (**C**,**D**). The images were obtained using a Leica DM750 microscope with a digital camera (100, 200 and 400× magnification).

Figure 6 presents PA with a canalicular-like pattern and a canalicular adenoma.

#### 3.1.2. Macroscopical Aspects

The varied macroscopic appearance of CPAs reflects the complex microscopic structure of this tumor, which is particularly evident on the cut surface, where solid, cystic, soft and white–yellow areas, as well as gelatinous and translucent areas with elastic or soft consistency, can be seen. CPAs are round–oval tumors, generally well delimited and frequently encapsulated, but the thickness of the capsule is variable and it may be partially or completely absent [16,17,19,30,31,35].

Less often, areas of hemorrhage or necrosis can be observed [34]. Also, in tumors developed from minor salivary glands, the capsule is not well developed or is even absent [16,17,31,33,35,52,55,58,62].

**Figure 7:**
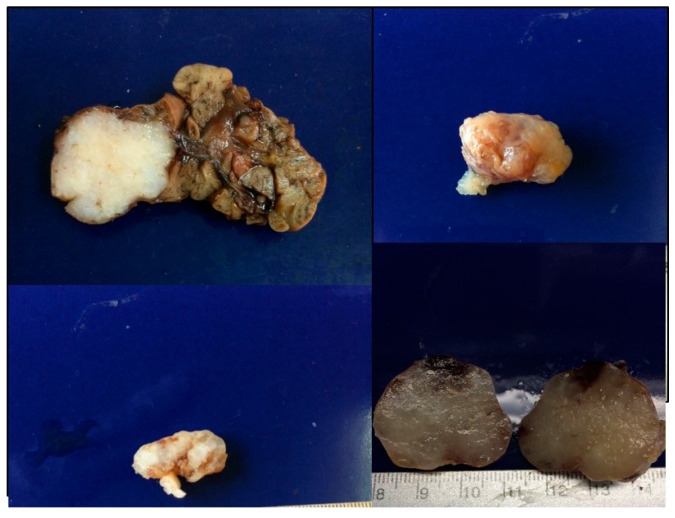
Typical gross features of PAs represented by white solid area (cut surface) and gelatinous, translucent areas.

Gross aspects of CPA are presented in Figure 7.

#### 3.1.3. Clinical Aspects

In most cases, PAs are single asymptomatic tumors, with diameters between 0.8 and 8 cm and slow growth—from 3 months to 10 years [16,17,18]. Some patients may present with discomfort when chewing, mild pain, and an increase in size 3 to 8 months before diagnosis [32,38,43].

PAs located in the palatine minor salivary glands may present clinically as submucosal masses, with slow growth and/or ulceration in some cases, pain and bleeding, symptoms considered to be associated with damage to the tumor surface during mastication [18,30,33,37,39].

Most cases of PAs located in the oral mucosa at the base of the tongue are asymptomatic, but after the tumor has grown in size patients complain of discomfort when swallowing [8].

Special attention should be given to rare PAs like those located in the trachea. These tumors of the tracheal wall are adjacent to the thyroid gland and can be interpreted on imaging examinations as thyroid tumors. Moreover, because tracheal PAs present with symptoms characterized by dyspnea and wheezing, they can be clinically misdiagnosed as bronchial asthma or chronic obstructive pulmonary disease [6,7].

#### 3.1.4. Differential Diagnosis of CPA

Benign salivary gland tumors that need to be differentiated from CPAs are basal cell adenoma, myoepithelioma, or Warthin tumors. PAs in which the stromal component predominates must be differentiated from soft tissue tumors such as extraskeletal chondroma and myxoma [16,17,64,66]. CPA must be distinguished from early carcinoma ex pleomorphic adenoma (CEPA). Atypical morphology in CEPAs is described as focal or diffuse changes or as multifocal carcinomatous areas, which replace the benign elements and are always accompanied by invasion and easily observed mitoses. Invasion in early CEPAs is classified as non-invasive, in situ, intratubular or intraductal tumors. In the invasive type of CEPAs the invasion mostly does not exceed 6 mm. Aspects suggestive for malignancy are considered to be rapid size growth and involvement of the facial nerve [17,81,82].

The newly described canalicular adenoma-like variant of PA should be differentiated from the canalicular adenoma of the salivary glands. In terms of location, this variant of canalicular-like PAs is localized in the major salivary glands, while canalicular adenoma is more frequently described in the minor salivary glands, especially those of the lip [78,79,80].

PA with lymphoid stroma must be differentiated from benign tumor lesions that present with lymphoid stroma such as Warthin tumor, and nonspecific chronic sialadenitis.

Of course, the essential aspect of CPA is represented by the typical morphology described. The absence of malignant cytomorphological features, frequent atypical mitoses and invasion are useful elements that help make the difference. The preservation of the lobulation of the salivary gland parenchyma, of the acini and the ducts is characteristic of an inflammatory lesion.

### 3.2. Atypical Aspects of PAs

#### 3.2.1. Microscopical Atypical Aspects of PAs

Case reports or retrospective studies that have analyzed PAs with atypical morphology reported in the English-language literature show that rare or atypical aspects can be found in both the epithelial and/or myoepithelial cellular component and in the stroma. Authors who classify PAs as belonging to unusual morphological categories either report isolated cases or retrospectively identify these features in the analyzed PAs [20,26,27,28,29,30,31,32,33,34,35,36,37,38,39,40,41,42,43,44,45,46,47,48,49,50,51,52,53,54,55,56,57,58,59,60,61,62,63].

Thus, in the cellular component, the presence of mucinous, squamous, lipomatous, osseous, endocrine-like or apocrine metaplasia is reported, with cells organized in solid or even cribriform-like areas [31,33,34]. Also, a study into 613 cases of PAs identified 301 cases of tumors with cells showing mitotic figures between 0 and 17 per high-power microscopic field and 25 cases in which even isolated atypical mitoses were present [31]. A case report described PA of the parotid gland in a 64-year-old woman that microscopically showed typical features described in these tumors, but also presented isolated atypical cells and aspects of vascular invasion of the epithelial component [32]. Cole GG and collaborators analyzed 23 PAs in which they identified and described epithelial cells with enlarged, irregular nuclei and prominent nucleoli, eight of the cases also presenting mitoses and six presenting aspects of tumor necrosis [34]. In none of these cases were features of malignancy reported, and the integrity of the tumor capsule did not influence the evolution of the cases with atypical adenomas [20,26,27,28,29,30,31,32,33,34,35,36,37,38,39,40,41,42,43,44,45,46,47,48,49,50,51,52,53,54,55,56,57,58,59,60,61,62,63].

The authors Cheuk W and Chang JK mention cell types that show more frequent malignant changes in PA, stating that: in the majority of cases (75%), luminal epithelial cells are the ones that show malignant transformation, while carcinomas described in the literature with myoepithelial or dual epithelial–myoepithelial differentiation (6–19%) are less aggressive [25].

PAs may be accompanied by areas of squamous metaplasia, sometimes with the formation of keratin pearls, both in the ducts and in the stromal tumor islands. Cystic formations filled with keratin and bordered by squamous epithelium have also been described. In PAs, aspects such as extensive inflammation and necrosis were observed, as consequences of spontaneous infarctions or as a result of fine needle aspiration. Within these tumors, numerous mitotic figures can be found, sometimes with cellular atypia, as well as areas of squamous metaplasia—characteristics that may be interpreted as malignant [47,54,55,56,57,58,59,60,61,62].

In the stromal component of PAs, foci of bone metaplasia or extensive hyalinization of the stroma can be observed, which may eclipse the epithelial component. Extensive hyalinization is reported especially in cases with prolonged evolution [16,17,68].

PAs with lipomatous-appearing stroma were infrequently identified [48,49,50,51]. The aspect where the lipocytic component is present in more than 60–70% of PA is considered to be a consequence of the accumulation of lipids in the myoepithelial cells or an aspect of lipomatous metaplasia of the myoepithelial cells [83]. However, tumors with a lipomatous component greater than 90% were named lipomatous PAs by Seifert G and collaborators [73,74]. A lipomatous component has also been reported in parotid PAs, in those that develop in the minor salivary glands of the oral mucosa and even those of the ceruminous glands [83,84,85,86]. In most studies, the existence of adipocytes in PAs has been considered a consequence of the metaplasia of myoepithelial cells into adipose cells, but some authors postulate it to be the result of a mechanical process. According to them, a lipomatous PA is the result of the isolation of adipocytes from adjacent tissues by the growing tumor [48,49,50,51].

Isolated studies describe aspects of vascular or neural invasion. In rare cases, isolated tumor cells or solid nests were identified in vascular spaces (both in the center of the tumor and at the periphery), this aspect being interpreted by some researchers as a consequence of the surgical intervention, while others suggest that it is evidence of the ability of PA to metastasize hematogenously [16,17,87].

The facial nerve can be affected in cases with parotid gland PAs. The evolution of patients with PA and perineural invasion has been monitored for periods between 9 and 32 months after surgery without observing any tumor recurrences for these tumors [26,27,32].

**Figure 8:**
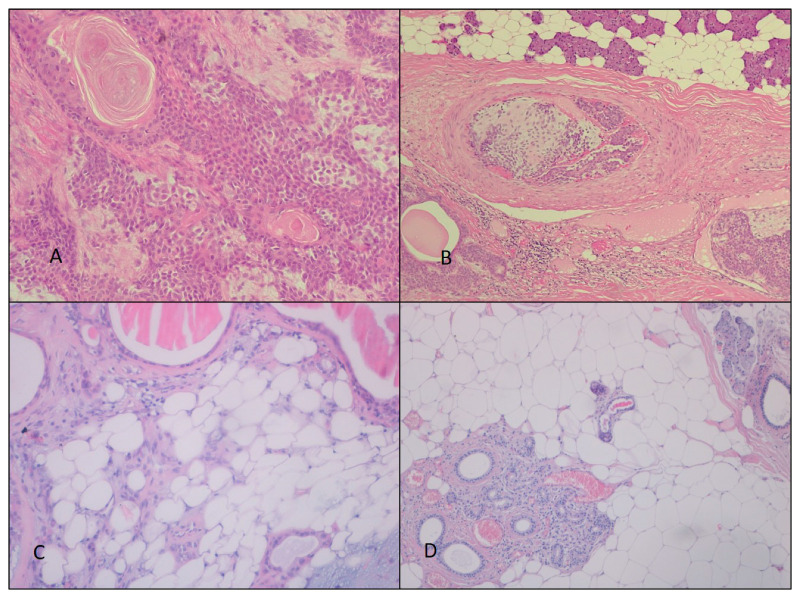
PA with squamous metaplasia (**A**), vascular invasion (**B**) and lipomatous stromal component (**C**). In the image (**D**) a lipoadenoma is presented. The images were obtained using a Leica DM750 microscope with a digital camera (200 and 400× magnification).

Atypical morphology in PAs is presented in Figure 8 with one of its mimickers.

#### 3.2.2. Atypical Macroscopic and Clinical Aspects of PAs

**Figure 9:**
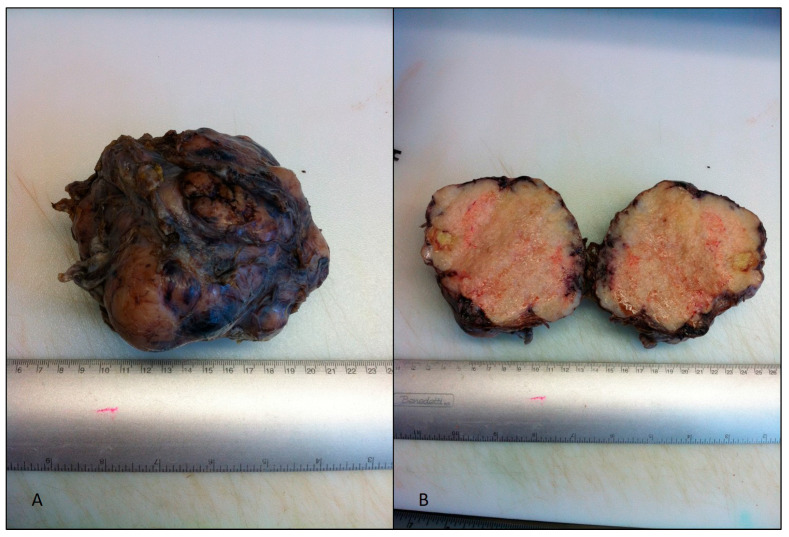
Giant PA—gross aspect, uncut (**A**) and on cross section (**B**).

An element of interest is the macroscopic and clinical aspect of PAs, which can be classified as unusual or atypical. The morphological features reported in these cases are those of large/giant CPAs with slow evolution over years. Thus, PAs with diameters ranging between 7 and 33 cm have been reported, which presented a slow, asymptomatic growth from weeks up to 30 years [18,30,33,37,38,39,40,41,42,43,44,45,46]. An example of giant PA is shown in Figure 9.

The majority of PAs are asymptomatic, but cases that were accompanied by discomfort in swallowing, dyspnea, surface ulceration, or pain have been reported. Pain was identified especially in those patients who reported an increase in tumor size in the last few months prior to diagnosis [36,37,44].

#### 3.2.3. Differential Diagnosis of Atypical PAs

The differential diagnosis of atypical PAs includes benign and malignant epithelial, myoepithelial or mesenchymal tumors of the salivary glands and soft tissues, as well as cystic and inflammatory lesions (Table 1). The aspects described as atypical for PA, such as atypical cells, metaplasia, aspects of invasion (vascular, perineural, into adjacent tissues or the capsule), those with necrosis and unusual dimensions, make the differential diagnosis of malignant tumors difficult [20,23,24,25,26,27,28,29,30,31,32,33,34,35,36,37,38,39,40,41,42,43,44,45,46,47,48,49,50,51,52,53,54,55,56,57,58,59,60,61,62,63]. The essential features of PAs include the admixture of ductal structures bordered by epithelial and myoepithelial cells, the chondromyxoid stroma with intermingled myoepithelial cells, and fibrous or hyalinized areas that are pathognomonic for the CPA type and helpful in differential diagnosis. Carcinomatous elements with a high mitotic rate (Ki-67 index higher than 5%,) and PA areas must be identified in the histopathological slide for a malignant mixed tumor diagnosis [82]. In addition, clinical features like rapid growth and facial nerve involvement are aspects that more frequently characterize a malignant tumor.

Knowing the morphology of lipomatous PA is also important. This type of PA should not be confused with a malignant tumor that invades or originates in the adipose tissue. To date, there are only a few studies that describe lipomatous PAs/PAs with lipomatous metaplasia published in the English language. In most studies, lipomatous lesions of the salivary glands are considered to be sialolipomas, lipoadenomas, lipomatosis and lipocytic metaplasia from pleomorphic adenoma. Sialolipoma is a primitive lipoma with areas of normal salivary tissue. Lipoadenoma is defined as a well-circumscribed nodule consisting of ductal proliferations without acinar differentiation or a myoepithelial component and adipocytes. Lipomatosis is a non-encapsulated, non-neoplastic lesion observed in patients with diabetes, liver cirrhosis, chronic alcoholism, or in the elderly where ductal elements or salivary acini are absent [48,49,50,51,83]. The fact that adipocytes are well differentiated, no lipoblasts are present, the lesion is encapsulated, there is no cytological atypia, necrosis or mitoses in the lipomatous stroma and it shows the morphological elements found in CPA confirms the diagnosis of lipomatous PA.

### 3.3. The Clinical Evolution of PAs

In most cases, PAs present as single tumors, but cases of multiple, synchronous or metachronous tumors, uni- or bilateral, in the same salivary gland or in different salivary glands, with identical or different morphology have also been reported [88,89,90]. The synchronous development of two major salivary gland PAs is rare, occurring in less than 2% of cases [91]. Multiple primary parotid gland tumors represent 1.7–5% of all salivary gland neoplasms [89]. Cases with double or triple association of three histologically different salivary tumors developed synchronously in the same salivary gland have also been reported [92,93,94,95,96,97,98,99]. Reports of PA with synchronous localization in the parotid gland and minor salivary glands are extremely uncommon [99]. Most cases described in the literature show synchronous tumors with different histological appearance, PA being most frequently associated with a Warthin tumor [90].

Studies commonly report that PAs have a favorable outcome after excision and rarely recur, mostly at 5 and 10 years after surgery for the initial primary tumor (in 3.4% and 6.3% of cases, respectively) [16,17,18,26,27,28,29,30,31,32,33,34,35,36,37,38,39,40,41,42,43,44,45,46,47,48,49,50,51,52,53,54,55,56,57,58,59,60,61,62,63,65,100]. The incidence of recurrence in PAs is between 0 and 17% [16,17,100]). In studies that analyzed atypical PAs, recurrences were reported in 13.32% of cases [31,47]. A series of studies have highlighted the fact that there are a number of factors that may be responsible for the development of these recurrences. These risk factors are: 1. enucleation of the tumor without safety margins, 2. damage to the tumor capsule with the discharge of mucinous content and the implantation of tumor cells, 3. the presence of adenomatous foci outside the tumor margins through the absence/penetration of the capsule (pseudopodia), 4. the abundance of myxoid stroma, 5. young age, 6. multiple tumors in the same gland, 7. tumors that extend beyond the zygomatic arch [25,101,102,103] or 8. situations where the tumor is multinodular [104,105].

Zinnis LOR et al. showed that there are cases with recurrent PAs that have more than one recurrence, indicating the fact that the percentage of developing new recurrences varies widely between 10 and 58% of the studied cases, estimating an average recurrence of 33.3% of cases for a follow-up period of 20 years [106]. Wittekindt C et al. conducted a study on 108 cases of PAs and reported tumor recurrences at 5 years in 42% and at 15 years in 75% of the studied cases [107]. The authors observed a higher rate of recurrences in females, young patients and those whose surgical treatment involved simple enucleation of the tumor, thus placing them in a category with a high risk for developing recurrences [107].

In cases with atypical PAs, no clear relation to a certain risk factor and the potential for relapse was identified, with follow-up of patients being performed for periods ranging from one month to 760 months after therapeutic interventions. One study reported that, of the 92 atypical PAs that relapsed, 42 had more than one relapse and one case became malignant [31].

In the majority of cases, tumor recurrences have the same histological structure as the initial tumor. There are also authors who believe that a high number of recurrences increases the possibility of malignancy of pleomorphic adenoma [25]. Therapeutic options in cases of recurrent PAs vary widely from simple patient follow-up, surgical reintervention and excision with facial nerve involvement to adjuvant radiotherapy. Radiotherapy is reserved for cases where the resection margins are affected—those with tumor capsule damage, or multiple or multinodular recurrences [31,100].

The malignant potential of PA has been investigated, raising many questions. Carcinoma ex pleomorphic adenoma, carcinosarcoma and MPA are types of mixed tumors of the salivary glands with malignant behavior and/or morphology [16,17,65]. Some authors have proposed the terms PA with areas of focal carcinoma or atypical pleomorphic adenomas for PAs with atypical/bizarre cells and mitoses [2,24]. There are authors who consider that PA with large areas of hyalinization and moderate mitotic activity (1.5/10 high power fields) have a 3.8% probability rate of transformation into a malignant mixed tumor [66]. In cases of PAs with long evolution, it has been shown that the risk of malignancy may increase; a tumor that has a rapid increase in size in just a few months after a long evolution can transform into carcinoma ex pleomorphic adenoma [31].

In general, the category of tumors classified as benign includes cellular proliferations that show isolated atypia and few mitoses, where invasion and metastases are absent. MPA belongs to a group of rare tumors that are histologically and molecularly identical to PA, but they metastasize [108]. PAs that have metastasized to the bone in the head and neck region and to the lungs are more frequently cited [81,82,108,109,110]. In case report studies, metastases have been identified in the cervical lymph nodes, oral mucosa, pharynx, skin, liver, retroperitoneum, abdominal wall, kidneys, mediastinum, pharynx, mammary gland, skull and central nervous system [81,82,109,110]. In most studies, MPAs have not shown any other aspect of malignancy apart from the potential to metastasize [110,111]. Usually, MPAs, like PAs, have been reported in female patients in their third and sixth decades of life, being predominantly located in the parotid gland [108]. After studying the data from the literature, we observed that this type of adenoma is present both in patients who have had tumor recurrences and in those where PA has not recurred [112,113]. The time from the diagnosis of PA to the identification of its metastasis varies widely between 1.5 and 55 years [81,82,109,110,111,112,113,114,115]. A fairly high mortality rate (22–40%) was observed in these patients [114,115,116,117]. Marioni G et al. classify these MPAs in the group of salivary gland tumors with low-grade malignancy [118]. Other authors suggested that this type of tumor may be an intermediate step for the transformation of PA into carcinoma ex pleomorphic adenoma [81,82,118,119, 120]. No cases of MPA were reported in the studies that analyzed atypical morphology in PAs.

**Figure 10:**
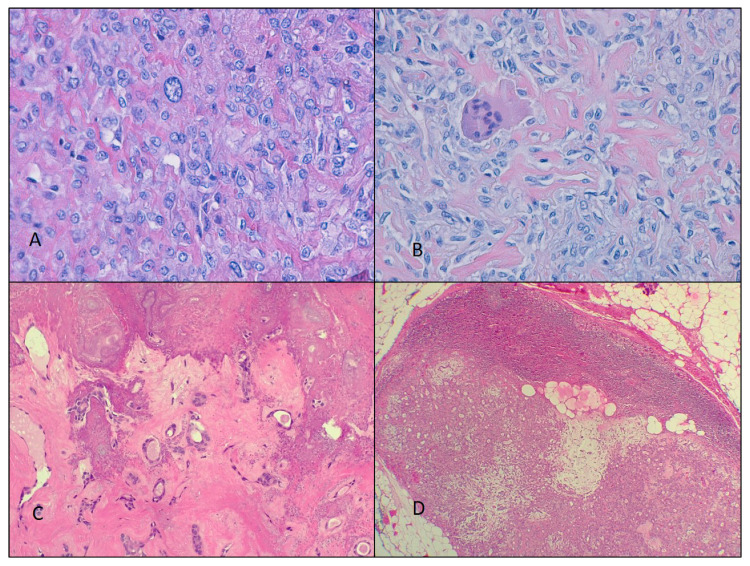
PAs with bizarre cells (**A**,**B**) and osseous metaplasia (**C**). In image (**D**) a metastatic PA in a lymph node is presented. The images were obtained using a Leica DM750 microscope with a digital camera (100, 200 and 400× magnification).

**Figure 11:**
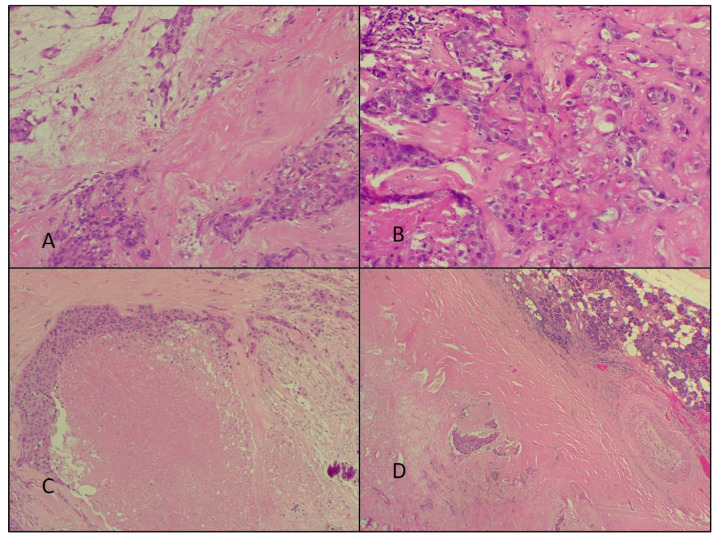
Case of CEPA with PA area (**A**), mucoepidermoid carcinoma (**B**) and salivary duct carcinoma (**C**,**D**) as malignant carcinomatous components. The images were obtained using a Leica DM750 microscope with a digital camera (100, 200 and 400× magnification).

Figure 10 illustrates atypical microscopic features of PA, including a case of lymph node metastasis of PA, highlighting the unusual biological behavior of these tumors. In Figure 11 a case of CEPA is shown to be compared with the atypical aspects identified in PAs.

## 4. Conclusions

The diversity of PAs refers to the variation in their morphological aspects. The aspects described as atypical for conventional PA, such as atypical cells, metaplasia, invasion, necrosis and unusual dimensions, are features that make it difficult to differentiate this benign tumor from malignant tumors of the salivary glands and soft tissues.

The microscopic appearance with identification of the pathognomonic aspects seen in PAs is the key tool used for diagnosis. The classic PA type is represented by both epithelial and myoepithelial cells arranged in structures with different patterns, accompanied by a typical chondromyxoid stroma. Aspects of metaplasia, especially squamous metaplasia, can be found both within the cellular component and in the tumor stroma of PAs. Extensive keratinization has also been described in these tumors. Atypical variants of PAs can show morphology and clinical features similar to malignant tumors, but their evolution is benign. Knowing the pathognomonic morphological aspects of PAs, as well as those atypical or more rarely reported elements in PAs, is crucial for avoiding an erroneous diagnosis followed by an unnecessarily aggressive treatment. Recognition of the lipomatous subtype of PA is not only important for differential diagnosis with salivary gland lesions with lipomatous component, but also for evaluating the relationship of the tumor with the adjacent tissues, as well as assessing the presence or absence of local invasion.

The study is focused on PAs with uncommon morphology and the challenges they create in the diagnostic process that can lead to missed, delayed or even incorrect diagnosis due to the rarity of reported cases. A lack of significant cellular atypia, rare and typical mitoses, and limited areas of necrosis are features that suggest a benign nature of proliferation.

## Figures and Tables

**Table 1:** Morphological aspects of PAs [20,26,27,28,29,30,31,32,33,34,35,36,37,38,39,40,41,42,43,44,45,46,47,48,49,50,51,52,53,54,55,56,57,58,59,60,61,62,63] and the differential diagnosis to consider.

PA Morphology	Main Differential Diagnosis
CPA—main features: epithelial/myoepithelial cells in chondromyxoid stroma (variable proportions)	Benign entities—epithelial component: basal cell adenoma, myoepithelioma, canalicular adenoma; stromal component: myxoma, extraskeletal chondroma Malignant: carcinoma ex pleomorphic adenoma
Lipomatous PA—main features: chondromyxoid stroma, epithelial/myoepithelial components, mature adipocytes (lipomatous component between 25 and 95%)	Benign entities: lipoadenoma, sialolipoma, interstitial lipomatosis, lipomatous atrophy Malignant: liposarcoma
PAs with bone formation—main features: mixture of epithelial/myoepithelial and chondromyxoid stromal components with identification of osseous tissue	Benign entities: chondromyxoid fibroma, extraskeletal chondroma, osteoma, osteoblastoma Malignant: low-grade chondrosarcoma
PAs with squamous metaplasia—main features: mixture of epithelial/myoepithelial and chondromyxoid stromal components with identification of squamous metaplasia or squamous cysts filled with keratin	Benign entities: salivary duct cyst, branchial cyst, dermoid cyst, chronic sialadenitis, necrotizing sialometaplasia, keratocystoma, salivary gland ischemia modification, Warthin tumor, ectopic thymoma Malignant: squamous cell carcinoma metastatic/invasive, epithelial–myoepithelial carcinoma, adenosquamous carcinoma
Atypical PAs—main features also useful in the differential diagnosis are: lack of significant cellular atypia, rare mitoses with a Ki-67 index < 5%, rarely encountered area of necrosis	

## Data Availability

No new data were created or analyzed in this study. Data sharing is not applicable to this article.

## Supplementary Materials

The following supporting information can be downloaded at: https://www.mdpi.com/article/10.3390/diagnostics16142168/s1, Table S1. Studies that explored atypical PAs.
