# Supplementary material for: Pleomorphic Adenomas and Their Atypical Morphology: Pitfalls in the Diagnosis of Salivary Gland Tumors
Source: Diagnostics (Basel). 2026 Jul 11;16(14):2168. doi: 10.3390/diagnostics16142168 (PMC13408538; doi:10.3390/diagnostics16142168)
Supplement: Supplementary file 1 [file diagnostics-16-02168-s001.zip › diagnostics-4396495-supplementary.pdf]

Study year	Age/Sex/Location	Nr. cases	Clinical features	Invasion vascular/neural/capsular	Gross aspect	Microscopy	Treatment	Follow-up
Gubod ER et al.2022	32/F/PG(angle of mandible)	1	Painless swelling, slowly growing in size for two years	No invasion	0.7/0.7 cm, nodule, light and tan	CPA with metaplastic woven bone formation	Surgery (excision of the tumor)	Two weeks, no recurrence
Alzumaili B et al.2022	12-86/216M, 397 F/561PG,34SG, 15msg, 3lg	613	US	452 showed capsular invasion/pseudopods	US (with median size of tumor nodule of 2.1 cm	Majority CPAs, with 213 had also sq m, 37 mm, 10 o m,81 encr-like atypia, mitoses in 301 (0-17 HPF) with 25 atypical mitoses	Surgical resection and in 39 cases received also radiotherapy	Median 216 (23-750 mth), 92 recurrences with 42 subsequent recurrences, one patient with malignant transformatio n
Ethunandan M et al.2006	42 and 64/F/PG	2	Discomphort for 6 mth and the other asymptomatic present for years	One with vascular invasion (42/F)	US	One case with CPA and vascular invasion, the other (64/F) with atypical cells	Surgery	32 and 28 mths without disease
Radhika T et al 2021	34/F/palate	1	One mth swelling, ulceration of the surface, mild pain	Incomplete capsule	2.3/1.5/1.7cm	CPA with hyalinization, plasmocitoid cells and cribriform-like areas	Surgery	US
Cole GG et al.2025	23-80/15M, 8F/22PG,3SG,3 pf, msg	29	US	US	1.1-6 cm	23 cases had pithelial cells with nuclear or cellular enlargement, hyperchromatic nuclei,	Surgery	0-12 years, 0 recurrences

						irregular nuclear contours, coarse chromatin, prominent nucleoli, anisonucleosis, 8 also mitoses, and 6 with tumor necrosis, 6 am		
Kondo T .2009	14/F/PG	1	asymptomati c slow-growing mass	Complete capsule	2 cm, yellow on cut surface	PA with 95% mature adipose tissue	Surgery	No recurrence (period of follow-up US)
Shi H et al.2008	70/M/ ms of soft palate and lateral pharyngeal wall and left retromandibular fossa	1	gradually progressive painless swelling in the throat	Well define margins, no infiltration	7/4/3cm, grey and white cut surface	CPA with om	Surgery	3 years, no recurrence
Tarsitano A et al 2015	83/M/PG	1	Slow-growing mass with 30 years evolution	Firm, multinodular, irregular mass	33/27/16 cm	CPA	Surgery	5 years, no recurrence
Salih AM et al.2018	49/F/PG with extension in the pf	1	swelling in the left side of the jaw for ten years which increased in size gradually over the last eight months	US	7/4/5 cm	CPA	Surgery	2 mths, no recurrence
Pareek YK et al.2022	30-81/4F, 10M/PG	14	Swelling, two patients presented	US	2-3.5 kg	Benign PA, US other	Surgery	Minimum 6 mts, no recurrence

			with ulceration on the overlying skin, evolution 5-20 years			morphological aspects		
Datta G et al.2025	31/F/PG	1	slow-growing, painless mass, evolution of two years after prior excision of PAC	Complete capsule	13/7/6 cm mass with globular outer surface, white, on cut surface white with hemorrhage areas	CPA	Surgery	7 days, no recurrence
Bordoy-Soto M.A et al.2016	45/M/ms palate	1	slow-growing, dysphagia and dyspnea, 17-years evolution	US	9/9/10 cm smooth surface,	CPA	Surgery	10 mths, no recurrence
Ajiya A et al.2025	67/F/PG	1	Slow-growing, painless, 10-years evolution	US	23/19/20 cm	CPA	Surgery	US
Prajapati BJ et al.2020	52/F/SG	1	Slow-growing painless swelling for the last 5 years that increased rapidly in the last 6 months	US	15/13 cm, cut surface solid grey-white	CPA	Surgery	No recurrence but the period of the follow-up was US

Benedetti A. et al.2021	56/F/SG	1	Painless mass, slow-growing for 22 years, associated with pain in the last 3 mths	US	7.52/5/4 cm in size	CPA	Surgery	One year follow-up, no recurrence
AlKindi M et al.2020	36/M/PG	1	Slow-growing swelling with 4 years evolution, no obvious symptoms	Partial capsule	7/13/7 cm bilobed and ovoid in shape	CPA with sq m	Surgery	7 years, no recurrence
Perumal CJ et al.2012	20/F/SG	1	painless swelling increasing in size over an 8-year period.	US	multinodular, nontender, hard, and mobile; size of 16 /15/12 cm	CPA	Surgery	US
Takahama A Jr et al 2018	78/M/PG	1	painless mass with 30 years evolution and superficial ulceration of the skin	US	Firm, multinodular; size-28/20/16 cm	CPA with hyaline stroma, squamous differentiation and plasmocitoid cells	Surgery	No recurrence but the period of the follow-up was US
Nokaneg E.N. 2022	28/F/ms palate	1	Asymptomatic swelling	US capsule but with invasion of the palate bone	3/4.9 cm, fluctuant mass	CPA	Surgery	Recurrent tumor after 4 years
Kimura TC.2026	8-83/102F, 69 M/142 PG,19 SG, 10 msg	171	mobile (21), asymptomatic (19),	PAs from PG and SG mostly encapsulated, tumors from msg partial or incomplete capsule; multinodular growth pattern,	0.5-12 cm, fibroelastic nodules (14), or multiple fibroelastic	CPA with sq m, cystic degeneration hyalinized or myxoid material	Surgery	23 had recurrences, the follow up period ranged from 3-360 mths

				with multiple, unencapsulated tumor nodules dispersed within fibrous connective tissue or adjacent salivary parenchyma in the recurrent cases	nodules (9 cases).			
Matsuzaka K et al. 2003	33/F/ms palate	1	slow growing painless mass	Encapsulated	3/2 cm, cut surface yellow	CPA with 80% of the tumor univacuolar adipocytes	Surgery	US
Vaishali AN et al 2020	30/M/ms upper lip	1	painless swelling, 8 mths evolution	Partial capsule	1.5 × 1 cm in diameter with cut surface smooth and yellowish in color	CPA with adipose tissue	Surgery	US
Musayev J et al.2014	32/F/ms palate	1	asymptomatic, slowly growing mass	US	2/1.8/14 cm in size, well-circumscribe, yellow-colored	CPA with 90% of the tumor univacuolar adipocytes	Surgery	US
Shah SS et al.2018	24/M/ms palate	1	painless, slowly growing mass with few weeks evolution	US	5/ 4 cm, oval-shaped	CPA, plasmacytoid cells and with 50% adipose tissue	Surgery	Two years follow-up, no recurrence
David A et al.2022	49/M/SG	1	5-year history of a painless submandibular swelling, slow-growing	Encapsulated	3.5/ 3/1.5 cm salivary gland with a 1 cm diameter solid cream white nodule that extended to the	CPA with nests of squamous cells. and cysts with central keratinization	Surgery	US

					resection margin			
Kaveri H et al.2014	35/F/ms palate	1	2-years history of a swelling that increased in size in the last 3 mths	US	5/5 cm, irregular mass	CPA with extensive sq m	Surgery	US
Goulart MC et al.2011	37/M/ms upper vestible	1	10-year history of a painless mass in the upper vestibule	Encapsulated	US	CPA with extensive sq m and keratin cyst formation	Surgery	Three years follow-up, no recurrence
Hosur MB et al.2024	37 and 14/ F and M/ ms palate	2	One case (37/F)painless swelling in the left posterior palatal region which had been slowly growing for 12 years; the other (14/M) painful swelling in the right posterior palate of 3 to 4 month duration	well-circumscribed, well-encapsulated	1.5-2.5 cm, grey and brown masses	CPA with plasmacytoid and epithelioid cells and keratin filled cyst	Surgery	US
Sharma S et al.2018	50/F/ms palate	1	Ulcerated swelling of the palate for the past 1 week	Encapsulated	grayish-white, firm; size of 2.6/ 2.9/1.7 cm3	CPA with florid squamous cells (50% of the tumor) arranged in	Surgery	1.5 years follow-up, no recurrence

						nests, islands or in sheets with or without extensive cyst		
Lin HP et al.2026	43/M/ms palate	1	Mass on the palate increasing for several mths	Partially encapsulated	nodule, measuring 0.7 cm in greatest dimension	CPA with sq m and cystic spaces filled with keratin flakes and evidence of lipomatous change	Surgery	US
Lim S et al.2013	32/F/ms palate	1	three-month history of painless mass on palate with bony erosion of maxilla	US	whitish yellow mass well-circumscribed with size of 3.5/3.0/2.0 cm	CPA with more than 45% of the epithelial cells represented by squamous cells with superficial and deep-seated keratin cysts	Surgery	no recurrence, 10 mths follow-up
Urs AB et al.2019	35/M/ms cheek	1	painless swelling over the right side of face since 4 years with recurrent cheek biting in the same area	Encapsulated	ovoid mass light brown in color, on cut surface white and focally yellow; 2/2/2.7 cm	CPA with mucin and keratin filled cysts	Surgery	under regular follow-up
Nishi T et al. 2015	22/M/ms cheek	1	painless swelling with 4 years evolution	US	grayish white soft tissue piece measuring 2/1.5 cm	CPA with sq m and formation of keratin pearls.	Surgery	no recurrence, 1 mth follow-up

Brisebois S et al. 2015	37/M/ms palate	1	US	US	well-delineated submucosal nodule of 1.7 cm in the greatest diameter	CPA massive sq m with keratinization	Surgery	no recurrence, 1-year follow-up
Singh A et al.2022	39/F/ms palate	1	soft tissue mass in center of hard palate since 6–8 years	Partially encapsulated	Grey and white mass with hemorrhagic areas and size 2/2.5 cm	CPA sq and adipose tissue	Surgery	no recurrence, 10 mths follow-up
Mayer M et al 2022	80/f/PG	1	3 mts visible evolution, osteodestructive	Poorly demarcated	Clear margins calcified deposits	CPA with osseous component	Surgery	no recurrence, 26 mths follow-up
Kunimoto Y et al.2013	55/F/PG	1	Subsequent PAs with metastatic PA in the gluteal region, now presenting PA invading temporal bone and facial nerve palsy	Invading internal auditory canal, the facial nerve	US	CPA	Surgery	no recurrence, 12 mths follow-up
Hogg GE et al.2020	55/M/PG	1	mass with 6 mths evolution, hard, immobile	Invasion the facial nerve and the mastoid bone	3/2 cm	CPA	Surgery	no recurrence, 9 mths follow-up

Table S1. Studies that explored atypical PAs [20,26-63]. Abbreviations: F=female. PG=parotid gland,CPA=classic (C) pleomorphic adenoma(PA) - type classic microscopic aspect; M=male; SG=submandibular gland;msg=minor salivary gland;lg=lacrimal gland; US=unspecified; sq m=squamous metaplasia; mm=mucinous metaplasia; o m=ossous metaplasia, encr-like=endocrine-like; HPF=high power field; mth=month; pf=parafaringian space; am=apocrine metaplasia
